# Beyond Global Models: Mapping the Spatially Contingent Relationship Between Soil Sand Content and Woody Invasion

**DOI:** 10.3390/life16050709

**Published:** 2026-04-22

**Authors:** Beatriz Sosa, David Romero, José Carlos Guerrero, Melina Aranda, Marcel Achkar

**Affiliations:** 1 Instituto de Ecología y Ciencias Ambientales, Facultad de Ciencias, Universidad de la República, Iguá 4225, Montevideo 11400, Uruguay; jguerrero@fcien.edu.uy (J.C.G.); achkar@fcien.edu.uy (M.A.); 2 Grupo Biogeografía, Diversidad & Conservación, Departamento Biología Animal, Facultad de Ciencias, Universidad de Málaga, Málaga, Campus Teatinos s/n, 29071 Málaga, Spain; davidrp@uma.es; 3 Cátedra de Dasonomía, Departamento de Producción Vegetal, Facultad de Agronomía, Universidad de Buenos Aires, Av. San Martin 4453, Ciudad Autónoma de Buenos Aires 1417, Argentina; maranda@agro.uba.ar

**Keywords:** spatial analysis, riparian systems, biological invasions, *Gleditsia triacanthos*, soil sand content, spatial structure, local statistics, classification and regression trees

## Abstract

Riparian ecosystems are being increasingly threatened by hydrological alteration and biological invasions, yet the role of local environmental heterogeneity in shaping invasion dynamics remains poorly understood. To address this, we tested the hypothesis that invasion patterns are spatially structured and therefore cannot be fully captured by global statistical models. We evaluated this hypothesis by analysing the relationship between soil sand content and the abundance of *Gleditsia triacanthos* in a riparian forest of the Esteros de Farrapos and Islands of the Uruguay River National Park, Uruguay. Generalized Linear Mixed Model revealed no significant relationship between soil sand content and *G. triacanthos* abundance (χ^2^ = 1.93, *p* = 0.17). In contrast, spatially explicit analyses showed that relationships between sand content and abundance were spatially contingent. Positive linear relationships predominated in areas with low sand content (mean 24.5%, *n* = 12), while negative relationships were restricted to the highest sand levels (mean 87.6%, *n* = 3). Intermediate sand-content zones (mean 47%, *n* = 16) showed no consistent patterns. These results suggest that invasion patterns vary across spatial contexts and may reflect the influence of different processes operating locally, indicating that relying solely on global analyses risks misinterpreting drivers and overlooking fine-scale variation. Our findings emphasize that understanding invasive species in heterogeneous systems requires considering whether mechanisms operate at local or broad scales, and that explicitly analyzing spatial structure can guide both hypothesis formulation and field study design.

## 1. Introduction

Riparian systems are highly threatened by a variety of factors that modify their water regimes, such as canalization, dredging, dam construction, irrigation systems, and water extraction [1]. In the context of global change, biological invasions pose an additional threat to these ecosystems [2,3]. Successful invasions in riparian systems are often associated with areas where the hydrological regime has been previously modified [4]. To guide the conservation of these vulnerable ecosystems, it is crucial to understand the relationships between ecological processes and abiotic conditions [5].

Riparian systems are characterized by spatial and temporal heterogeneity resulting from the redistribution of sediments, organic matter, and other materials. Deposition and erosion processes are key drivers of vegetation development [6]. These processes operate at the local scale [7] and spatially structure soil texture [8]. Changes in soil texture are linked to environmental conditions and water dynamics: smaller particles (light textures) are deposited in areas with lower flow rates, which leads to longer flooding periods and reduced infiltration, while coarse-textured soils occur in high-energy zones with faster flow and shorter flooding periods [9]. Despite their ecological relevance, the effects of soil texture on vegetation in riparian systems are not yet fully understood.

Evidence from different systems shows contrasting effects of sediment characteristics on vegetation. In riparian forests associated with intermittent watercourses, deposition processes negatively affect the richness, diversity, and occurrence of woody species [10]. Similarly, in Nigerian wetlands, sand content limits the development of invasive plant species [11]. Conversely, in saline wetlands, sediment supply positively affects vegetation growth [12], and in temperate flooded grasslands, sediment deposition facilitates the invasion of the herbaceous species *Phalaris arundinacea* [13]. Although these systems differ in ecological context, they share underlying sedimentary and hydrological processes that structure soil texture and vegetation dynamics making them relevant for understanding potential responses in riparian forests.

Riparian landscapes are characterized by strong geomorphological heterogeneity produced by fluvial processes, generating spatial variation in sediment deposition and soil properties. Such heterogeneity can influence the establishment and spread of woody species, potentially producing spatially structured invasion process. Although some invasive species exhibit broad environmental tolerance, local environmental gradients may still influence their spatial distribution within heterogeneous landscapes. When ecological relationships vary across space, global statistical models (which estimate a single average effect) may obscure localized responses to environmental gradients. We therefore hypothesize that invasion processes in this riparian system are spatially structured and cannot be fully characterized using global statistical models alone. Based on this premise, we propose that: (1) global models may fail to detect a consistent association between soil sand content and the abundance of *Gleditsia triacanthos* across the study area, and (2) spatially explicit analyses will reveal localized zones where these variables are related indicating a spatial structured invasive process. Assessing this point would evaluate the relevance of scale-sensitive frameworks for understanding woody species invasion in highly dynamic and heterogeneous systems, such as riparian forests.

## 2. Materials and Methods

### 2.1. Study Area

**Figure 1:**
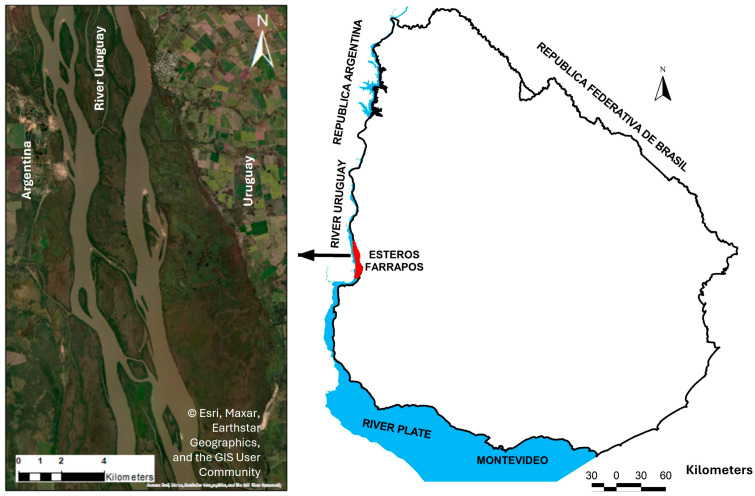
Location of the study area along the western bank of the Uruguay River, Uruguay. The red polygon indicates the boundaries of the Esteros de Farrapos e Islas del Río Uruguay National Park. The satellite image displays the continental area of the protected area. Source: Esri; data providers: Maxar, Earthstar Geographics, and the GIS User Community.

The study area is located in the riparian forest of the Esteros de Farrapos and Islands of the Uruguay River National Park, Río Negro Department, Uruguay (32°37′36.308″ S, 58°09′40.920″ W) (Figure 1).

The National Park comprises a fluvial wetland located in the final section of the Uruguay River, where a decrease in flow rate forms a zone of active deposition with fluvial islands and sandbanks. It covers an area of 21565ha, including lakes, sandy beaches, floodable grasslands, and riparian forests [14].

The fluvial bank on which the riparian forest develops is characterized by a landform that presents different shapes that can be described as ridges, spurs, valleys, hollow and flat [15]. In this area, the riparian forest is of biogeographic importance due to the intrusions of the subtropical forests of the Paranaense region through the Uruguay River, which have their southern limit of distribution in this area [16,17]. The mean annual temperature and precipitation in this area are 18 °C and 1244 mm [18], respectively. Among the main native plant species in the study area are *Phyllanthus sellowianus* Müll., *Sebastiania schottiana* (Müll. Arg), *Ruprechtia laxiflora* Meisn., *Sapium haematospermum* Müll. Arg., *Pouteria gardneriana* (A.DC.), *Guettarda uruguensis* Cha & Schltdl., *Combretum fruticosum* Stuntz, *Terminalia australis* Cambess., *Salix* sp., *Inga vera* Wild., *Eugenia mansonii* O. Berg. and *Eugenia uruguayensis* Cambess [19]. The main human activities carried out in the study area are cattle ranching and beekeeping; logging and hunting are less frequent [20]. The main threats to biodiversity conservation are the invasion of *Gleditsia triacanthos* and the erosion of coastal habitats [20]. This erosion process seems to be mainly driven by hydrological processes [21].

### 2.2. Invasive Species

*Gleditsia triacanthos* is a woody species belonging to the Fabaceae family, which is native to North America, particularly the Mississippi River basin [22]. It is tolerant of a wide range of environmental conditions; however, it shows only moderate tolerance to flooding [23] and reduced regeneration under dense forest canopies [24]. The species has high invasive potential due to a combination of specific reproductive traits, including both asexual and sexual reproduction, rapid fruiting, high seed production and germination rates, and a short juvenile stage [25]. Vegetative reproduction occurs mainly via coppicing and resprouting from stumps and roots, generating clonal individuals that promote persistence and local expansion following disturbance events [26]. Dispersal occurs through hydrochory and endozoochoric, primarily mediated by cattle [27].

It is recognized as an invasive species in a wide variety of regions and environments. It has an invasive status in Spain [28], Australia [29], Serbia and Ukraine [30], Argentina [31,32] and Uruguay [33]. In Uruguay, it is one of the woody species with the greatest potential to displace native forests [33]. In the riparian forests of the Esteros de Farrapos National Park, the invasive process of *G. triacanthos* has displaced almost all native woody species in some areas.

### 2.3. Sampling Design

Biological invasions progress through distinct stages that vary in space and time [34]. In the study system, the invasion of *G. triacanthos* has been previously characterized based on spatial analysis of invasion patterns comprising two zones: a consolidated area and a propagation area representing the active invasion front [35]. This zonation reflects different spatiotemporal phases of the invasion process. In the present study, we focus on the propagation area, where abiotic constraints such as soil sand content are expected to play a stronger role in regulating spread; this expectation underlies its selection and defines the spatial extent of our analysis.

Transects were established within the propagation area defined in a previous study [35], which determines the spatial extent of the present analysis. Within this extent, transects were spaced at an average distance of 550 m, a resolution previously shown to be suitable for zoning the spatial distribution pattern of *G. triacanthos* at the study site [35]. This sampling resolution was selected to detect broad-scale patterns relevant to invasion propagation while limiting the influence of fine-scale local heterogeneity. Consequently, the resulting sample size provides an ecologically meaningful dataset that balances statistical reliability with the scale of the invasion process.

**Figure 2:**
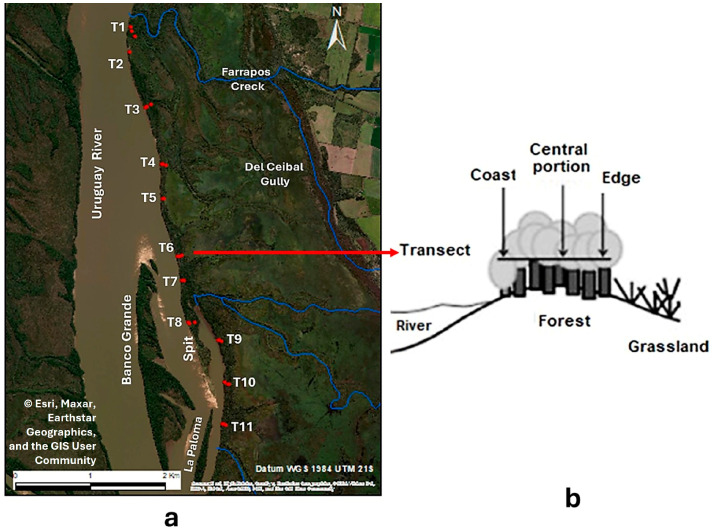
Sampling design. (**a**) Location of the sampling transect within the riparian forest along the Uruguay River (T1–T11). Sampling plots (red points) are distributed along each transect. (**b**) Schematic representation of plot arrangement along each transects, extending from the river margin (**left**), through the forest interior, to the forest–grassland edge (**right**). Source: Esri; data providers: Maxar, Earthstar Geographics, and the GIS User Community.

A total of 11 transects were established, each planned to include three 4 × 20 m plots spanning the coast edge, forest center, and forest–grassland transition (Figure 2). Plot size was defined following a previous study that characterized the spatial distribution pattern of *G. triacanthos* and delineated consolidated and propagation areas [35] as it corresponds to the sampling unit at which variation in abundance was resolved in that analysis. Due to access difficulties, two plots could not be sampled, resulting in a total of 31 plots. All adult individuals of *G. triacanthos* within the sampled plots were counted. In this study, adult trees in the plots were counted as individual plants as we did not distinguish between genets and clonal ramets. Although this approach avoids direct inference about recruitment, observed abundance may reflect a combination of clonal expansion and independent establishment, both potentially influenced by soil conditions. Regardless of origin, adult individuals contribute to propagule production and thus to invasion dynamics.

The spatial position of each plot was recorded using a Garmin 60CSx GPS (Garmin Ltd., Olathe, KS, USA). The field work was performed in December 2017 during an average hydrological regime since no exceptional floods or drought events were registered.

Soil sampling was carried out using the composite method [36]. Within each plot, a zig-zag path was delimited where the point of extraction of the sample was marked at regular intervals of 1.30 m starting at one of its ends. Prior to the extraction of each subsample, the vegetation cover or leaf litter was removed from each point [36]. Within each plot, a total of 15 subsamples were taken from the first 10 cm of soil. The different subsamples were deposited together in a container where they were mixed. A 500 g sample was collected from this mixture in a polyethylene bag. As a result, a total of 31 samples were collected (one sample per plot). The samples were processed at the Soil, Plant and Water Analysis Laboratory at the Alberto Boerger Experimental Station, La Estanzuela, of the National Institute of Agricultural Research (January 2018) using the Bouyoucos method. This procedure consists of soaking the soils in a 5% Calgon solution for 15 to 20 h and then dispersing them with a soil mixer running at a speed of about 16,000 r.p.m. for 2 min [37]. Field data are available [38].

### 2.4. Overview of the Analytical Framework

To address the study hypothesis, we implemented a hierarchical analytical framework combining a confirmatory global model with exploratory spatial analyses. The framework was designed to first test whether a consistent global relationship exists between environmental conditions and the invasive process, and then to explore how this relationship varies across the landscape.

The analysis proceeded in four steps. First, a generalized linear mixed model (GLMM) was used as a confirmatory test to evaluate whether a single, consistent association exists between soil sand content and *G. triacanthos* abundance across the study area. Second, spatial variability in both variables was characterized using wavelet analysis, identifying locations of abrupt spatial change and providing context for subsequent analyses. Third, local spatial patterns were quantified using Getis–Ord Gi statistics and interpolated to generate continuous surfaces for descriptive interpretation. These surfaces were then analyzed using classification and regression trees (CARTs) and spatialized in a GIS environment to identify zones with similar combinations of environmental conditions and species abundance. Finally, the robustness of the resulting spatial patterns was evaluated by examining their ecological coherence and potential over-zonation effects.

### 2.5. Global Modelling of the Relationship Between G. triacanthos Abundance and Soil Sand Content

First, to assess the relationship between soil sand content and the abundance of *G. triacanthos* without accounting for spatial structure, we fitted a generalized linear mixed model (GLMM). The analysis was conducted in R, version 4.5.2 [39]. The model was built using sand content and *G. triacanthos* abundance data from each plot (*n* = 31). Using the fitdistrplus package (version 1.1-11) [40], we verified that abundance data followed a negative binomial distribution, which is characteristic of count data with overdispersion (variance greater than the mean). The model was fitted using the glmer.nb function from the lme4 package (version 1.1-35) [41], with *G. triacanthos* abundance as the response variable, sand content as the fixed effect, and transect as a random factor. Transect was included as a random effect to account for non-independence among plots within the same transect, to respect the sampling design, and to control for unmeasured spatial heterogeneity while focusing inference on the relationship between sand content and *G. triacanthos* abundance. Finally, we used the Anova function from the car package (version 3.1-2) [42] to obtain the Analysis of Deviance Table (Type II Wald chi-square tests), and the plot_model function from the sjPlot package (version 2.8.16) [43] was used to visualize the predicted relationship.

### 2.6. Spatial Variability of G. triacanthos and Soil Sand Content in the Study Area

To characterize spatial variability in *G. triacanthos* abundance and soil sand content, we applied wavelet analysis, which allows for the detection of spatial patterns as a function of position and scale along a one-dimensional transect [44]. This approach identifies locations where abrupt changes occur, providing a spatially explicit description of heterogeneity.

We focused on position variance, where peaks indicate locations with sharp changes relative to neighboring values and can be interpreted as spatial transitions (edges) in the variable of interest [45,46]. Mean values of abundance and sand content per plot were used as input, and variance peaks were interpreted as locations of abrupt spatial change along the study area.

We used the Haar wavelet due to its sensitivity to sharp discontinuities and suitability for detecting edges [47,48]. Analyses were conducted using PASSaGE 2 [49], with a scaling factor of 1 and a maximum scale set to 10% of the transect length. No significance tests were conducted for the detected variance peaks; therefore, results from this analysis should be interpreted as a descriptive characterization of spatial variability rather than as statistically inferential.

General methodological overviews of wavelet analysis are provided in [50,51].

### 2.7. Mapping the Spatial Structure of the Relationship Between G. triacanthos Abundance and Soil Sand Content

To detect the spatial structure of the relationship between the abundance of *G. triacanthos* and soil sand content, we integrated local statistics, interpolation techniques, classification and regression trees, dispersion analysis and geographic information systems.

### 2.8. Local Statistics

Getis and Ord [52] introduced the local statistics Gi and G* to detect pockets of spatial association. Using these statistics, it is possible to identify spatial clusters of large and small attribute values [53]. Lately, these statistics were redefined as a standard variate by taking the statistic minus its expectation, divided by the square root of its variance; this new form increases these statistics’ flexibility and therefore their usefulness [54].

The standardized G* is essentially a Z-value and can be associated with statistical significance. In this context, high positive G* values indicate clusters of high values, whereas high negative G* values indicate clusters of low values. Values close to zero suggest a random spatial distribution [55].

In this study, high values of G* refer to areas where the high abundance of *G. triacanthos* is surrounded by other areas of high abundance, and alternatively low values of G* are identified in areas where low values of abundance are close to each other. To detect the spatial relationship between *G. triacanthos* abundance and soil sand content, we used the standardized G* statistic obtained from the hotspot analysis for the variables sand content and abundance of *G. triacanthos*.

Hot Spot Analysis (Getis–Ord Gi*) was performed using a fixed distance band. A distance-based neighborhood definition is appropriate when data are regularly distributed in space [56]. The distance threshold was defined as the minimum distance required to ensure that each feature has at least one neighbor. While this criterion is independent of sampling scale in its definition, the resulting neighborhood structure depends on the spatial configuration of the data, and therefore its interpretation is constrained by the sampling resolution. In our work, smaller thresholds would result in some observations having few or no neighbors, leading to unstable or undefined Gi* estimates. Conversely, larger thresholds would incorporate more distant observations, producing a smoother spatial signal that may obscure local spatial contrasts.

Because Gi* values are subsequently used as inputs for interpolation and CART analysis, the selected threshold effectively defines the scale at which spatial structure is analyzed. It therefore represents a balance between statistical stability and the preservation of spatial variability supported by the sampling design.

### 2.9. Interpolation Techniques

A mosaic structure is the simplest way to represent the spatial heterogeneity of ecological processes [57]. Thus, to map the spatial relationship between *G. triacanthos* abundance and soil sand content, we built a mosaic with 10 m × 10 m cells. In this study, we specifically used standardized Gi* values derived from soil sand content and *G. triacanthos* abundance. These statistics capture localized patterns of association rather than raw values, making them particularly suitable for visualizing spatial relationships in a continuous form. Notice that the accurate of interpolated values of local statistics at the latticed locations was previously reported [58].

We used the Inverse Distance Weighted (IDW) method, which is appropriate for regularly distributed data [59]. Importantly, IDW is an exact interpolator [60] and thus it preserves the calculated Gi* values at the sampling locations. This approach transforms discrete point statistics into a continuous spatial “signal,” providing the necessary input for the subsequent classification and regression tree (CART) partitioning. The interpolated surface reflects spatial continuity inferred from observations and is constrained by the sampling resolution; consequently, it cannot resolve fine-scale spatial structure and may smooth transitions, particularly near boundaries.

Interpolation (IDW) and Hot Spot analysis were performed with the geoprocessing tools available in ArcGIS Version 10 software (ESRI Inc., Redlands, CA, USA).

### 2.10. Classification and Regression Trees

To characterize the spatial structure of the relationship between the abundance of *G. triacanthos* and soil sand content, we applied a regression tree approach, which is commonly used to analyze relationships between variables in mosaic-like spatial structures [61]. Interpolated standardized G* values for soil sand content were used as the independent variable, and interpolated standardized G* values for *G. triacanthos* abundance were used as the dependent variable.

Regression trees identify increasingly homogeneous subsets of the data by recursively partitioning the predictor space into nodes that minimize within-group variability in the response variable [62]. Once a split is defined, it is not reconsidered in subsequent steps, and the partitioning process continues until a stopping criterion is reached [63].

The tree was built using the entire dataset to preserve the full spatial context of the invasion front as the limited number of independent sampling plots would not support a traditional data split without likely compromising the spatial signal required to identify localized ecological associations. The CRT (classification and regression tree) growth method was used, and tree depth was limited to five levels to constrain model complexity. The resulting structure was not accepted as a final result but was internally validated by evaluating its spatial coherence against known geomorphological features and analyzing the relationship between node size and correlation strength to detect potential over-zonification (see Section 2.11 and Appendix B).

All analyses were conducted using SPSS V.9 software.

### 2.11. Geographical Information Systems and Dispersion Analysis

In this study, each terminal node of the classification tree groups’ observations with similar combinations of interpolated standardized G* values and retains their associated geographic coordinates. When a node encompassed spatially disjoint areas, it was subdivided according to the number of distinct geographic zones represented.

To characterize the relationship between soil sand content and *G. triacanthos* abundance within each node, we evaluated the association between variables using scatterplots and fitted linear and second-order polynomial models. Based on these fits, relationships were classified as positive, negative, polynomial, or absent.

These relationship types were subsequently spatialized to produce a zonation map of the study area, where each zone represents a distinct form of association between soil sand content and *G. triacanthos* abundance.

Because of the methodological workflow, there is a potential risk that some splits reflect method-induced spatial structure rather than stable ecological relationships. To evaluate the robustness of the resulting zonation, we assessed model behavior at multiple levels. First, we examined the ecological coherence of higher-level (parental) nodes, which capture the dominant spatial structure of the system (Appendix B, Appendix B.1). Second, we analyzed the relationship between node size and correlation strength (r values) to evaluate potential overfitting and over-zonification at finer spatial scales (Appendix B, Appendix B.2). Accordingly, the resulting zonation is interpreted as a robust, scale-dependent representation of spatial heterogeneity within the constraints of the adopted methodological framework, rather than as a fully parameter-invariant solution.

## 3. Results

### 3.1. Relationship Between G. triacanthos Abundance and Soil Sand Content

**Figure 3:**
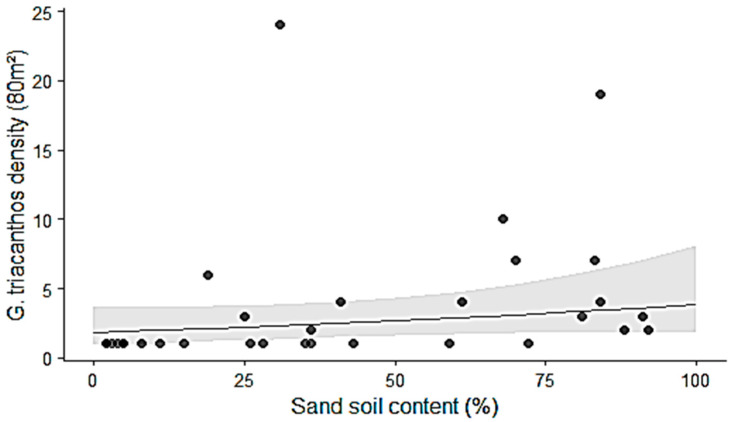
Abundance of *Gleditsia triacanthos* (number of individuals per 80 m^2^) as a function of soil sand content. Points represent observed data from the plots (n = 31), while the line shows the model-predicted relationship. The shaded area corresponds to the 95% confidence interval.

The analysis of deviance from the generalized linear mixed model indicated that the relationship between *G. triacanthos* abundance and soil sand content was not significant (χ^2^ = 1.93, *p* = 0.17) (Figure 3).

### 3.2. Spatial Variability of G. triacanthos and Soil Sand Content in the Study Area

**Figure 4:**
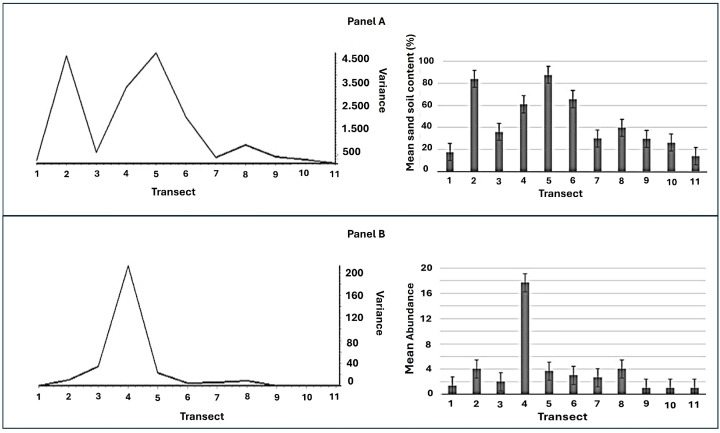
Local spatial variation in soil sand content (Panel **A**) and *Gleditsia triacanthos* abundance (Panel **B**) along the 11 study transects. Left panels illustrate localized variability (position variance) derived from a Haar wavelet transform analysis. Right panels show mean values (±SE) for each variable per transect.

Wavelet analysis using the Haar function revealed variance values for soil sand content ranging from approximately 0.8 to 4.3, with two major peaks at Transects 2 and 5 (variance of 4.3 and 4.2, respectively), consistent with abrupt changes (edges) in sediment texture in the study area (Figure 4 Panel A).

For *G. triacanthos* abundance, variance values ranged from approximately 0.0 to 200, with a single prominent peak at Transect 4 (variance 200), indicating a sharp spatial transition in species abundance (Figure 4 Panel B). This peak is located about 550 m north of the first major sand content variance peak.

### 3.3. Detection of the Spatial Relationship Between G. triacanthos Abundance and Soil Sand Content

**Figure 5:**
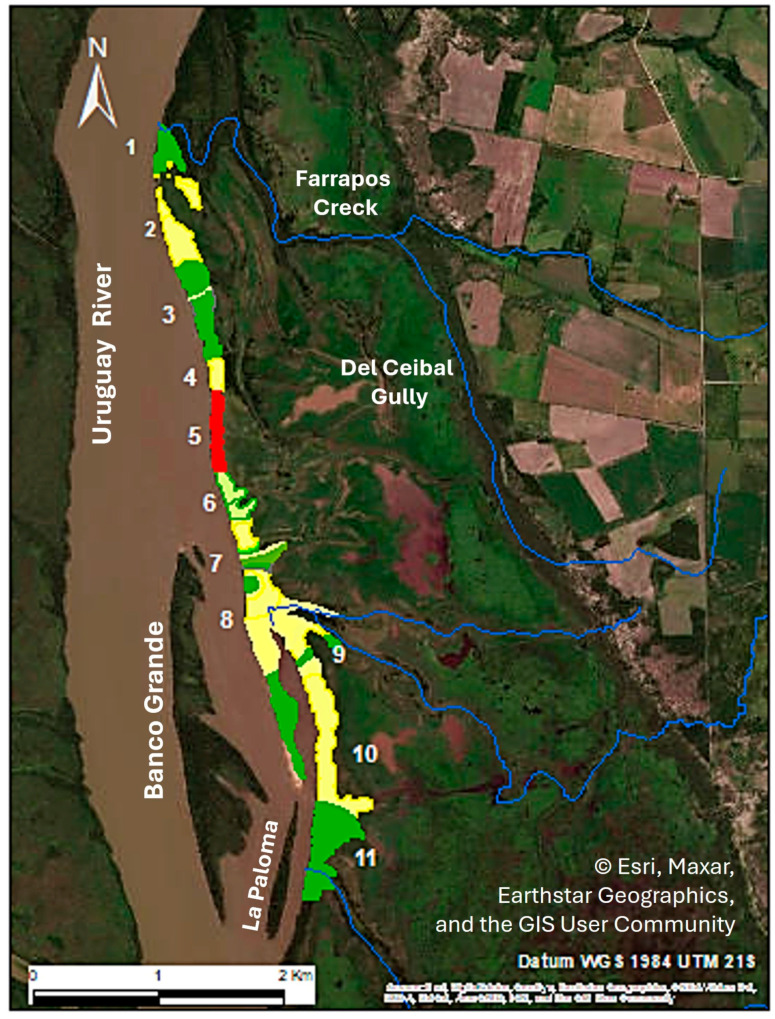
Relationship between soil sand content and *G. triacanthos* abundance in the propagation area based on the 51 spatialized sub-nodes. Green: areas with a positive linear relationship; Light green: areas with a polynomial relationship; Yellow: areas with no detectable relationship; Red: areas with a negative linear relationship. Numbers 1–11 indicate the transect positions along the invasion front. Source: Esri; data providers: Maxar, Earthstar Geographics, and the GIS User Community.

The classification tree detected a total of 36 nodes (see Appendix A) with a risk estimate (within node variance = 0.22). The spatialization of the 36 statistical nodes resulted in 51 distinct sub-nodes to account for geographically non-contiguous areas. Dispersion analysis of these 51 units identified 6 sub-nodes with a negative linear relationship, 24 with a positive linear relationship, 19 with no detectable relationship, and 2 fitting a second-order polynomial (see Table A1, Appendix B.2). The spatial arrangement of these 51 sub-nodes enabled the final zonation of the study area (Figure 5).

Based on the 31 sampling plots, positive linear relationships were associated with low sand content (mean 24.5% *n* = 12), no relationship occurred at intermediate sand levels (mean 47%, *n* = 16), and negative linear relationships were restricted to the highest sand content levels (mean 87.6 *n* = 3).

The robustness of the resulting zonation was evaluated through analyses of node coherence and potential overfitting, as detailed in Appendix B.

## 4. Discussion

Our results show that, when spatial variability is not considered, analyses fail to detect a significant relationship between soil sand content and the abundance of *G. triacanthos*, suggesting that a consistent global relationship was not detected. However, the spatial descriptive analysis indicates that associations between sand content and abundance are concentrated at the extremes of the sand gradient. This pattern is consistent with the behaviour of broadly tolerant species but suggests that local environmental conditions may influence abundance under specific contexts, a nuance that is not captured by global-scale analyses. Ignoring these spatial patterns could lead to incomplete interpretation of potential drivers and limit the effectiveness of management strategies. In this context, geomorphic processes are considered a potential source of spatial variability in soil sand content across the study area.

Zones characterized by high sand content are spatially structured across the study area and spatially coincide with geomorphic features associated with depositional dynamics. Increases in sand content observed at transects 2 and 4–6 occur in areas consistent with active sediment deposition, suggesting localized zones of sediment transport and reworking. In contrast, the reduction in sand content at transect 7 may be related to depositional processes occurring upstream, particularly in front of Banco Grande island, potentially influencing sediment availability at the sampled location. A subsequent increase in sand content at transect 8 coincides with the position of the spit sand bar, where deposition processes are likely to operate at the transect scale. Toward the southern transects, the progressive decrease in sand content may reflect spatial variation in sediment deposition within the spit zone, possibly associated with reduced local sediment availability and flow energy. Overall, these patterns suggest that local variation in soil sand content is associated with the relative position of transects within the geomorphic configuration of the system.

Negative relationships between sand content and *G. triacanthos* abundance were detected only at the highest levels of sand content, corresponding to areas associated with depositional dynamics and potentially higher disturbance. Under these conditions, the observed pattern is consistent with processes such as sediment instability affecting seedling establishment and early survival, suggesting that sand content may influence abundance at the extreme of its gradient. In contrast, no relationship was detected across most intermediate-sand-content zones, including the core area of invasion, indicating that under these conditions, sand content is not consistently associated with variation in abundance.

Positive relationships between sand content and *G. triacanthos* abundance exhibited a more spatially complex pattern. The clearest positive associations were observed at the lowest extremes of sand content (Transects 1 and 11), where conditions may favour establishment. At intermediate sand levels, these associations were limited to specific locations, such as Transect 3 (near the invasion core) and the transition area represented by Transects 7–8. This latter area, situated between the continental margin and the spit system, represents a transition zone recognized for its ecological relevance [64]. The observed patterns suggest that this transition zone may serve as a key spatial context for the invasion’s development toward the spit system, where higher densities were recorded. Furthermore, the positive relationship detected at the southern extreme of the spit is consistent with early stages of biogeomorphological succession, where vegetation establishment and sediment dynamics are often intrinsically linked [65]. Collectively, these results reveal a spatially structured mosaic of associations that is inconsistent with a single global trend, providing support for our hypothesis that local ecological signals are often obscured by spatially averaged analyses. These variations suggest that the relationship between sand content and abundance is likely driven by different ecological and geomorphological mechanisms operating across the landscape. The specific processes driving these relationships require further experimental evaluation to be fully confirmed.

Our findings suggest that sand content acts as a spatially contingent ecological filter. Importantly, the core area of high *G. triacanthos* abundance occurs in zones where no relationship with sand content was detected. This raises a key ecological question: are invasion dynamics within this hotspot driven by broader-scale factors such as propagule pressure, disturbance regimes, or historical establishment, or do local conditions facilitate the invader’s success independently of soil texture? Addressing this question requires explicitly linking invasion processes to the spatial structure of environmental heterogeneity. The zonation derived from our spatially explicit analyses provides a descriptive framework for identifying where different invasion mechanisms may be expressed across the landscape. Mechanisms operating at broader spatial scales would be expected to manifest across multiple zones, whereas locally operating processes may be more evident within specific spatial contexts.

Landscape positions can differ in their influence on ecological processes, with certain locations and spatial configurations acting as key areas for maintaining ecosystem structure and function; the Ecological Security Pattern (ESP) framework has been proposed as a way to identify and prioritize such areas [66]. The relevance of this approach is reinforced by recent developments in ESP frameworks that incorporate multiple management scenarios to identify priority areas under changing conditions [67]. Previous work in the study system has developed a spatiotemporal model (SWIRS) that simulates the spread of *G. triacanthos* under different management scenarios [68]. Integrating the zoning approach presented here with this modeling framework provides a way to link spatial structure with scenario-based dynamics, supporting the identification of priority areas for intervention within a spatially explicit context. This integration highlights how spatial zoning can contribute not only to ecological interpretation, but also to decision-oriented landscape management.

Despite the potential of the proposed zonation for improving our understanding of the invasion dynamics of *G. triacanthos*, several limitations should be acknowledged. For instance, quadratic relationships between sand content and invader abundance were only detected in two nodes; while these may reflect localized ecological complexity, they may also result from methodological artefacts associated with fitting non-linear responses in spatially heterogeneous settings. Similarly, some areas showing positive relationships can be interpreted considering their geomorphological context or proximity to the invasion core, but the possibility of overestimating these responses cannot be excluded. Therefore, a central challenge is the identification of an optimal sampling intensity that is sufficient to capture meaningful ecological heterogeneity without introducing the statistical artifacts associated with high spatial autocorrelation. While our *n* = 31 captured the primary spatial structure of this relationship, future research should focus on determining when a spatial zonation has reached sampled saturation.

While our analysis identifies sediment texture as an important variable associated with the spatial distribution of *G. triacanthos*, invasion dynamics may also be influenced by other factors, such as nutrient availability. Recent studies in riparian aquifers highlight that ecological processes can be governed by a hierarchy of drivers, where broader-scale structures may override fine-scale heterogeneity [69]. In this context, a key limitation of our approach is the potential mismatch between the spatial scale at which environmental variables operate, and the sampling resolution used to characterize them. Our sampling design captures variation in sand content at a relatively coarse spatial grain but may not resolve other-scale drivers such as nutrient availability. This reflects a general methodological challenge in heterogeneous systems, where multiple processes operating at different scales cannot be simultaneously captured within a single sampling framework. Furthermore, although the resulting zonation is consistent with the landscape’s physical structure, its sensitivity to methodological decisions has not been formally evaluated. Additional analyses would be required to assess the stability of these spatial boundaries.

One key limitation of this zoning is that it relies on a single dataset, which restricts its ability to account for temporal heterogeneity. While episodic changes in soil sand content driven by sediment deposition or hydrological events could influence the observed patterns, the spatial context of the study area suggests that some geomorphological features may persist beyond a single sampling period. Visual inspection of historical imagery (2012–present, Appendix C) indicates that prominent depositional features, particularly in high-sand zones, have remained relatively stable over multiple years. In this context, the identified zonation can be considered a spatial template for future research. Using this template, temporal replication can be directed toward evaluating whether these localized patterns persist under different hydrological conditions. This approach provides a basis for moving from the description of spatial patterns at a single point in time toward understanding how spatially structured processes interact with temporal variability in riparian systems.

## 5. Conclusions

Our results indicate that the relationship between soil texture and *G. triacanthos* is not spatially uniform but varies across the landscape, forming a spatially structured mosaic. By moving beyond global models, the spatially explicit approach used here reveals that associations between sand content and abundance differ in strength and direction depending on spatial context. This suggests that, in highly heterogeneous riparian systems, average trends may obscure localized ecological signals, highlighting the need for scale-sensitive analytical approaches.

The relationship zonation developed in this study provides a framework for evaluating ecological drivers within their spatial context, supporting a transition from landscape-scale generalizations toward a more spatially explicit understanding of invasion processes. In this sense, the zonation can serve as a spatial reference for future research, facilitating the integration of additional variables and temporal dynamics within a consistent spatial framework.

From a management perspective, this approach can support the identification of areas where environmental conditions are more likely to constrain or facilitate invasion, providing a basis for prioritizing interventions within a spatially explicit context.

## Figures and Tables

**Figure A1:**
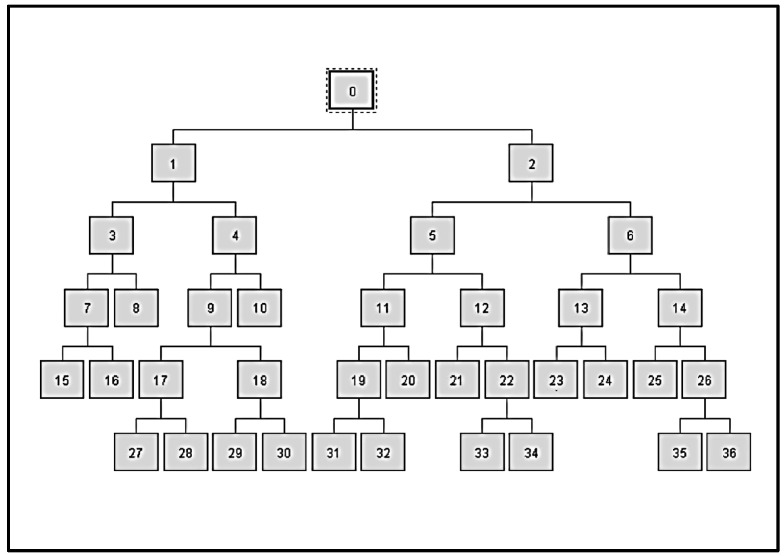
Regression tree schema.

**Figure A2:**
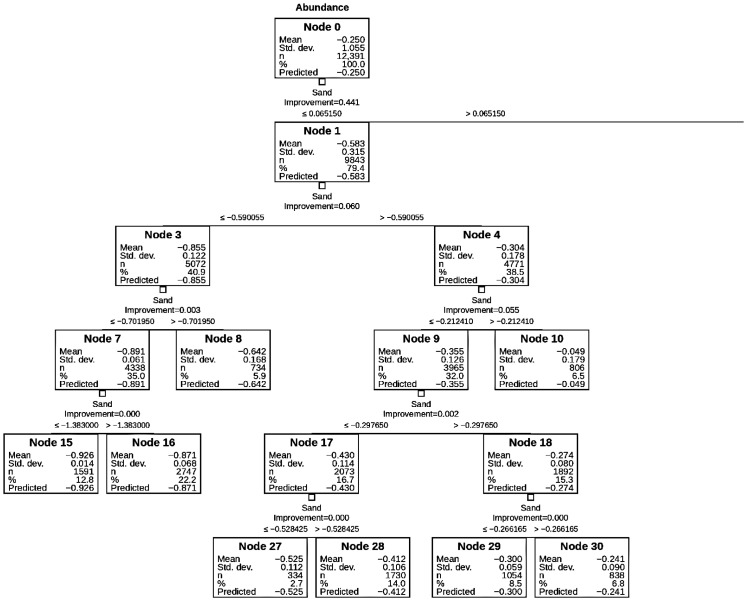
Details of regression tree resulting from Node 1.

**Figure A3:**
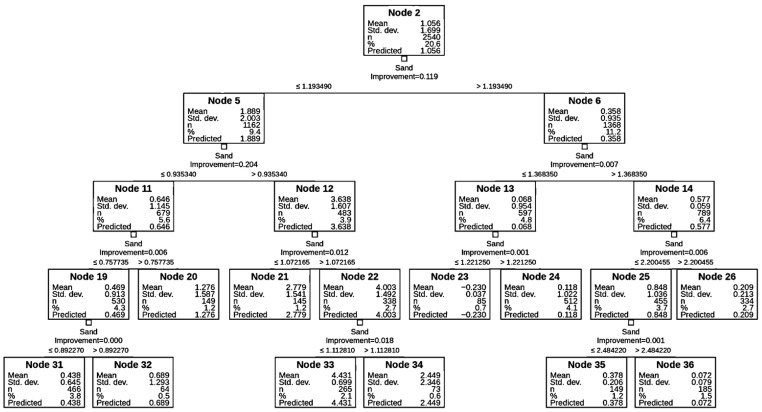
Details of regression tree resulting from Node 2.

**Figure A4:**
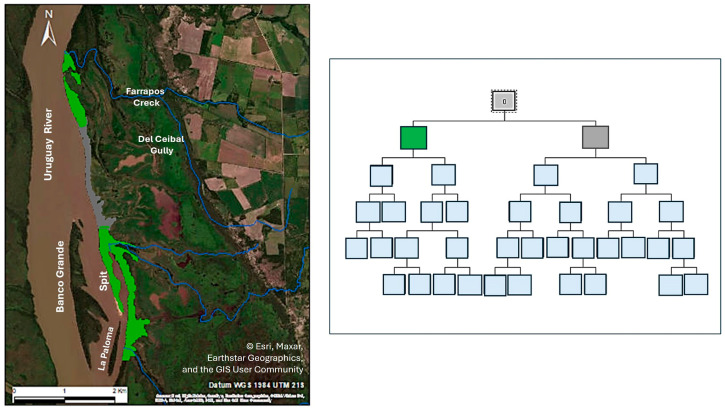
Primary hierarchical structure used to evaluate robustness of CART. Left main geomorphological zones were derived from parental nodes. Right. CART tree scheme. The color of each parental node indicates the color of the zone that derives from it. Source: Esri; data providers: Maxar, Earthstar Geographics, and the GIS User Community.

**Figure A5:**
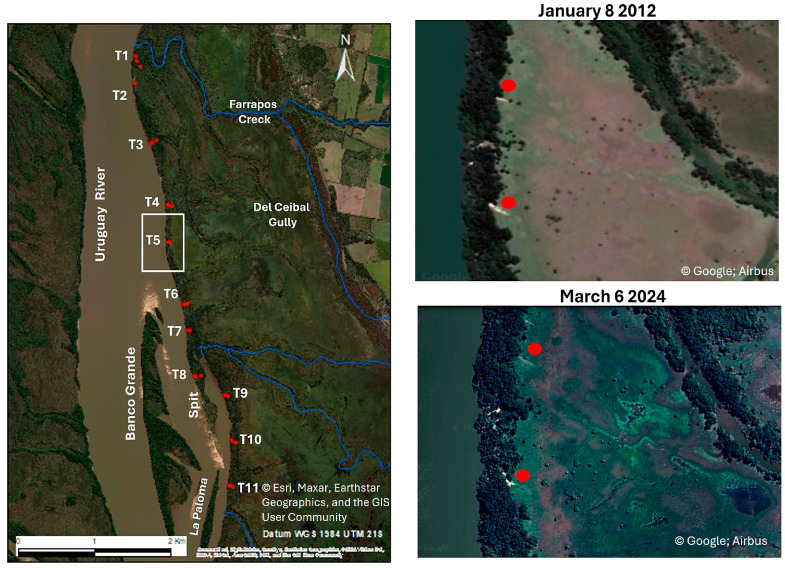
Comparison of active depositional areas in the surroundings of the zone with the highest soil sand content (Transect 5). The left panel shows a regional view of the study site with the location of transects. Source: Esri; data providers: Maxar, Earthstar Geographics, and the GIS User Community. The right panel presents Google Earth satellite imagery from 2012 and 2024, illustrating the persistence of depositional features through time. Red markers indicate areas of active deposition. Source: Google Earth; © Google; data providers: Airbus.

## Data Availability

Field data are available at https://redata.anii.org.uy/dataset.xhtml?persistentId=doi:10.60895/redata/HIUOWR (accessed on 1 April 2026).

## Appendix A. Classification and Regression Tree

In this Appendix, we present the results from the classification and regression tree analysis used to examine the relationship between soil sand content and the abundance of *Gleditsia triacanthos*. To aid in visualizing these results, we provide three figures: the first figure displays a diagram of the regression tree to illustrate its structure, while the following two figures present the statistical description for each node.

## Appendix B. Robustness Assessment and Over-Zonification Diagnosis

Classification and regression tree (CART) models are known to be susceptible to overfitting when excessive partitioning produces numerous small terminal nodes that capture noise rather than general patterns. To asses this risk, we first evaluated the ecological coherence of patterns obtained at higher-level parental nodes and subsequently examined the relationship between node size (number of pixels) and correlation strength (r values).

### Appendix B.1. Zonation at Parental Nodes

The spatialization of the two main nodes provided a first zonation of the study area consistent with its geomorphological structure (Figure 1). The first zone, in the northern part of the study area, is a floodplain connected to the Uruguay River via a discontinuity in the seasonal island, where active deposition occurs along the coast. The second zone is the narrowest section of the seasonal island, which is developing without major discontinuities and in direct contact with the main riverbed. The third zone begins near Banco Grande Island and includes the spit, which together influence sediment supply to the continental seasonal island.

### Appendix B.2. Analysis of Detected Relationship in Each Zone

Across all subnodes derived from the spatialization of the 36 nodes resulting from the CART analysis, 37% showed no detectable relationship between sand content and *Gleditsia triacanthos* abundance. Negative linear relationships were identified in 12% of subnodes, while quadratic relationships were rare, occurring in only 4%. Positive linear relationships were observed in 47% of subnodes. Importantly, both large and small zones were represented among areas with no detectable relationship as well as among those with positive relationships. Relationships were therefore not consistently associated with the smallest spatial units, indicating that the CART analysis does not systematically impose spurious associations.

**Table A1:** Results of the dispersion analysis for each spatialized node. Relationship types are indicated as follows: * negative linear relationship; ** negative quadratic relationship; *** no relationship detected; **** positive linear relationship. Node indicates the original node identified by the CART analysis, and Subnodes represent the number of spatial zones derived from the spatialization of each node. R indicates the strength of the relationship, Line shows the fitted equation, and N of pixels represents the number of pixels included in each zone.

Node	Subnodes	R	Line	N of Pixels
36	1	0.84	* y = −1.5765x + 4.1105	185
35	2	0.9586	* y = −2.5906x + 6.7188	55
		0.9806	* y = −0.5588x + 1.5542	96
25	2	0.9934	* y = −3.1401x + 7.8686	128
		0.9855	** −0.6319x^2^ + 2.4913x − 2.1112	328
24	3	0.9231	* y = −3.5505x + 8.4807	34
		0.8729	** −13.573x^2^ + 36.506x − 24.464	33
		0.1287	*** y = −0.017x − 0.2333	254
23	1	0.092	*** y = 1.6224x − 2.1953	86
34	2	0.9651	* y = −4.8295x + 10.024	43
		0.6458	**** y = 0.0521x − 0.321	32
33	1	0.0002	*** y = −0.3998x + 4.9596	261
21	2	0.967	**** y = 5.3245x − 1.859	116
		0.74	**** y = 0.0497x − 0.3168	30
32	2	0.9832	**** y = 3.5315x − 0.1465	23
		0.0138	**** y = −0.0465x − 0.2378	41
31	2	0.9972	**** y = 2.9303x + 0.2185	221
		0.7398	**** y = 0.0746x − 0.318	245
30	4	0.0275	*** y = 0.0176x − 0.359	117
		0.994	**** y = 2.4746x + 0.2652	261
		0.811	**** y = 0.0718x − 0.3198	103
		0.7191	*** y = 3.1549x + 0.5545	1158
29	6	0.0872	*** y = 2.171x + 0.3092	1054
		0.5114	*** y = 2.2161x + 0.2072	47
		0.0683	*** y = 0.0672x − 0.3222	14
		0.0244	*** y = 0.6348x − 0.071	160
		0.8684	**** y = 1.672x + 0.343	19
		0.1457	*** y = 1.9155x + 0.2525	138
28	5	0.597	*** y = 1.1297x + 0.0632	203
		0.9785	**** y = 2.9622x + 0.456	567
		0.5221	*** y = 0.3556x − 0.1719	592
		0.9968	**** y = 1.6723x + 0.3435	115
		0.5487	*** y = 0.764x − 0.0932	259
27	3	0.0636	*** y = 0.9993x − 0.0109	71
		0.1482	*** y = 0.1081x − 0.294	91
		0.8898	**** y = 1.7321x + 0.379	78
		0.9276	**** y = 1.9138x + 0.4634	68
		0.9667	**** y = 2.2002x + 0.6856	26
10	4	0.0016	*** y = −0.0963x − 0.0614	807
		0.9913	**** y = 2.503x + 0.2669	196
		0.7797	**** y = 0.0814x − 0.3196	84
		0.8508	*** y = 2.367x + 0.4335	451
15		0.8143	**** y = 0.1604x − 0.6851	1585
16		0.8208	**** y = 0.2163x − 0.5222	605
		0.917	**** y = 0.591x − 0.3332	405
		0.0858	*** y = 0.0349x − 0.8755	1756
		0.9789	**** y = 0.2657x − 0.5282	111
8	4	0.8837	**** y = 0.1042x − 0.2975	170
		0.4655	*** y = 0.8862x − 0.1362	180
		0.9891	**** y = 2.0541x + 0.5448	198
		0.9483	**** y = 2.1251x + 0.6412	40

## Appendix C. Active Depositional Zones Through Time

This Appendix provides qualitative temporal context for the geomorphic structures underlying the zoning presented in the main text. Because the spatial analysis is based on a single sampling period, it does not explicitly capture temporal variability in sediment dynamics. To assess whether the main depositional features associated with zones of contrasting sand content represent transient conditions or relatively stable landscape elements, we examined historical Google Earth imagery from 2012 to the present. The year 2012 corresponds to the earliest image available with sufficient visibility to enable consistent comparison of geomorphic features through time.

Attention was given to the comparison of active depositional areas in the surroundings of Transect 5, where the highest soil sand content was recorded. Visual inspection indicates that these depositional features have remained consistently expressed across multiple years. This observation supports interpretation of the spatial patterns identified in the zoning.
